# Screening for Novel LRRK2 Inhibitors Using a High-Throughput TR-FRET Cellular Assay for LRRK2 Ser935 Phosphorylation

**DOI:** 10.1371/journal.pone.0043580

**Published:** 2012-08-28

**Authors:** Spencer B. Hermanson, Coby B. Carlson, Steven M. Riddle, Jing Zhao, Kurt W. Vogel, R. Jeremy Nichols, Kun Bi

**Affiliations:** 1 Primary and Stem Cell Systems, Life Technologies Corporation, Madison, Wisconsin, United States of America; 2 The Parkinson’s Institute, Sunnyvale, California, United States of America; Hertie Institute for Clinical Brain Research and German Center for Neurodegenerative Diseases, Germany

## Abstract

**Background:**

Mutations in the leucine-rich repeat kinase-2 (LRRK2) have been linked to Parkinson’s disease. Recent studies show that inhibition of LRRK2 kinase activity decreased the level of phosphorylation at its own Ser910 and Ser935, indicating that these sites are prime targets for cellular readouts of LRRK2 inhibition.

**Methodology/Principal Findings:**

Using Time-Resolved Förster Resonance Energy Transfer (TR-FRET) technology, we developed a high-throughput cellular assay for monitoring LRRK2 phosphorylation at Ser935. LRRK2-Green Fluorescence Protein (GFP) fusions were expressed in cells via BacMam. Phosphorylation at Ser935 in these cells is detected using a terbium labeled anti-phospho-Ser935 antibody that generates a TR-FRET signal between terbium and GFP. LRRK2 wild-type and G2019S are constitutively phosphorylated at Ser935 in cells as measured by TR-FRET. The phosphorylation level is reduced for the R1441C mutant and little could be detected for the kinase-dead mutant D1994A. The TR-FRET cellular assay was further validated using reported LRRK2 inhibitors including LRRK2-IN-1 and our results confirmed that inhibition of LRRK2 can reduce the phosphorylation level at Ser935. To demonstrate the utility of this assay for screening, we profiled a small library of 1120 compounds. Three known LRRK2 inhibitors were identified and 16 hits were followed up in the TR-FRET and a cytotoxicity assay. Interestingly, out of the top 16 hits, five are known inhibitors of IκB phosphorylation, two CHK1 and two CDC25 inhibitors. Thirteen hits were further tested in a biochemical LRRK2 kinase activity assay and Western blot analysis for their effects on the phosphorylation of Ser910, Ser935, Ser955 and Ser973.

**Conclusions/Significance:**

We developed a TR-FRET cellular assay for LRRK2 Ser935 phosphorylation that can be applied to the screening for LRRK2 inhibitors. We report for the first time that several compounds such as IKK16, CHK1 inhibitors and GW441756 can inhibit LRRK2 Ser935 phosphorylation in cells and LRRK2 kinase activity *in vitro*.

## Introduction

Parkinson’s disease (PD) is a progressive neurodegenerative disorder that affects 1% of people over age 60 and more than 5 million people worldwide. PD results primarily from the selective loss of dopaminergic neurons in the substantia nigra. Current available therapies only address the symptoms of PD, and the most effective treatment has been in use for more than 50 years. The identification of familial mutations associated with PD has presented novel targets for potential therapeutic development. Mutations in leucine-rich repeat kinase 2 (LRRK2) have been linked to autosomal familial and sporadic PD [1], [2] and LRRK2 has recently been pursued as a potential therapeutic target for PD [3], [4], [5].

LRRK2 is a large multi-domain protein that is phylogenetically related to the Receptor Interacting Kinase branch of the tyrosine kinase-like family of the human kinome. Indeed the kinase domain does bear homology to MAP3K kinases. It contains several potential protein-protein interaction domains including N-terminal ankyrin repeats, leucine-rich repeats and a C-terminal WD40 domain. Surrounded by these domains is the catalytic core containing a GTP-binding Ras of complex protein (ROC) GTPase domain, a carboxy-terminal of Roc (COR) domain and a serine/threonine kinase domain [2]. The pathogenic mutations have been identified to be located mostly in the catalytic core of LRRK2. Mutations in the ROC GTPase domain (R1441C, R1441G, R1441H) and the COR domain (Y1699C) are reported to reduce the GTPase activity in the *in vitro* studies [6], [7]. The most frequent PD associated LRRK2 mutation encodes a glycine-to-serine substitution at residue 2019 (G2019S), within the conserved “DFG” motif of subdomain VII in the activation loop of the kinase domain. This mutation has been shown to increase kinase activity in several reports [5], [8]. The enhanced GTPase and kinase activities have been linked to neuronal toxicity in cultured cells [9], [10], [11], [12] as well as in the *in vivo* models [4]. Inhibition of LRRK2 kinase activity is shown to protect against LRRK2-induced toxicity both *in vitro* and *in vivo* [4], [13], indicating the therapeutic potential of LRRK2 kinase inhibitors. To date, several compounds have been reported to have inhibitory activity on LRRK2. These include the fairly selective compounds LRRK2-IN-1 [14], TAE684 [15] and CZC-25146 [13] and several non-selective compounds such as sunitinib, H-1152 [16], indirubin-3-monoxime, SP600125 and GW5074 [4].

LRRK2 is found to be phosphorylated at multiple sites throughout the protein and can be classified as autophosphorylation sites or sites of constitutive phosphorylation, which are detected on active LRRK2 expressed in cells. Multiple autophosphorylation sites on LRRK2 have been mapped [17], [18] and at least one has been utilized as a specific output of LRRK2 activity experimentally [19]. LRRK2 has also been shown to be phosphorylated at multiple sites including Ser910, Ser935, Ser955, Ser973 and Ser976 [20], [21], [22]. Phosphorylation at these sites is hypothesized to be carried out by cellular kinases in a potential feedback phosphorylation loop that is dependent on LRRK2 kinase activity [16], [20]. Phosphorylation at the two conserved residues Ser910 and Ser935 is required for binding of LRRK2 to 14-3-3 isoforms, which seems to regulate the cellular localization of LRRK2. Some pathogenic mutations such as R1441C display decreased phosphorylation of these two residues, thereby disrupting the interaction of LRRK2 and 14-3-3, leading to the accumulation of LRRK2 within cytoplasmic inclusions [21]. Acute inhibition of LRRK2 kinase activity leads to dephosphorylation of Ser910 and Ser935, which disrupts the 14-3-3 binding and alters cytoplasmic localization of LRRK2. These results suggest that monitoring the phosphorylation status of Ser910 and/or Ser935 could provide a mean to evaluate LRRK2 kinase inhibitors and pathway biology. We are unaware of a LRRK2 autophosphorylation site that has been shown to be responsive to acute LRRK2 inhibition in cell culture.

Several biochemical assay formats have been reported and utilized for measuring *in vitro* LRRK2 kinase activity. These include a high-throughput screening (HTS) compatible Time-Resolved Förster Resonance Energy Transfer (TR-FRET) assay using either LRRKtide or Nictide as the substrate [5], [23], [24], standard radioactive enzymatic assay using purified or immunoprecipitated LRRK2 (truncated or full-length) [14], and kinase binding assays [25], [26]. These assay formats have enabled the discovery of compounds with inhibitory activities against LRRK2 kinase. A chemical proteomics approach was also reported that led to the identification of selective LRRK2 kinase inhibitors such as CZC-25146 [13]. For the measurement of LRRK2 cellular kinase activity, commonly used methods include Western blot analysis of autophosphorylation or phosphorylation of LRRK2 at Ser910 and Ser935 in cells [4], [14], [16], [20]. Neurite outgrowth/retraction and TUNEL assays have been used to measure LRRK2-mediated toxicity in neuronal cells [10], [13]. These cellular assays are limited in terms of throughput and assay workflow. Here, we report the development of a high-throughput compatible homogenous LanthaScreen® TR-FRET cellular assay for the measurement of LRRK2 Ser935 phosphorylation and its application in the screening for LRRK2 inhibitors.

## Results

### LRRK2-GFP Expression via BacMam Gene Delivery System

**Figure 1:**
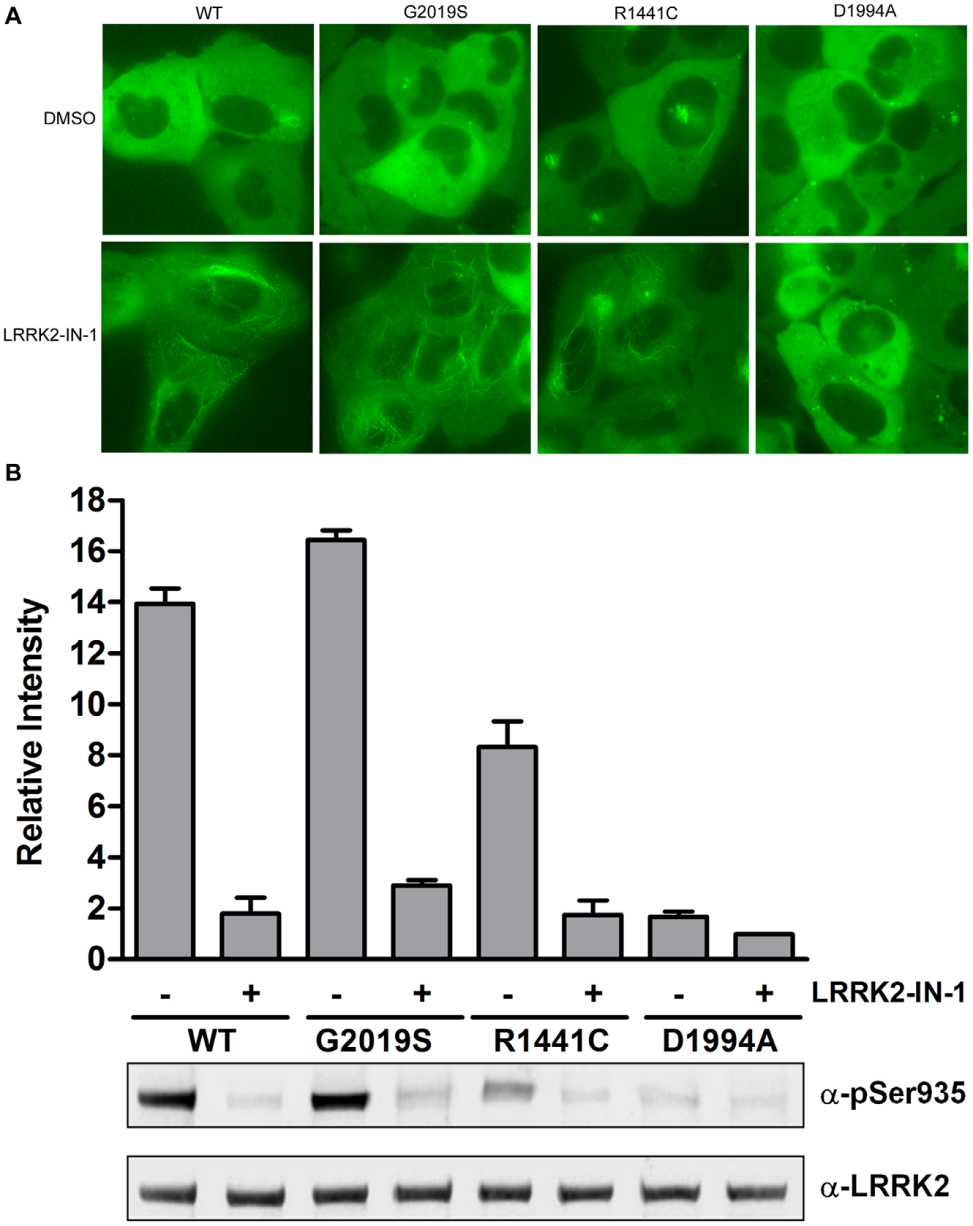
Expression of LRRK2-GFP in U-2 OS cells via BacMam gene delivery. U-2 OS cells were transduced with 20% BacMam LRRK2-GFP wild-type, G2019S, R1441C or D1994A. Cells were treated with DMSO only or LRRK2-IN-1 (3 µM) for 90 min. (A) GFP images were captured and representative images are shown. (B) Western blot analysis with anti-pSer935 antibody or anti-LRRK2 antibody. The quantifications are from 3 independent experiments.

The first step for developing a LanthaScreen® TR-FRET cellular assay is to generate cells expressing substrate of interest fused to GFP which acts as the fluorescence acceptor from a terbium labeled modification-specific antibody [27]. Here, GFP is fused to the C-terminus of full-length human LRRK2 wild-type, G2019S, R1441C and kinase-dead D1994A. We chose BacMam expression vectors as our vehicle to deliver LRRK2 due to the reproducible transduction and transgene expression of this large target in multiple cell types. U-2 OS cells were transduced with these BacMam reagents and analyzed by fluorescence microscopy and immunoblot analysis. Consistent with previous reports on the localization of N-terminal tagged GFP-LRRK2 [14], [21], [28], the C-terminal tagged LRRK2 wild-type, G2019S and D1994A mutants are expressed in the cytoplasm mostly in a diffused pattern with occasional aggregates observed in a small number of cells (Figure 1A). R1441C displayed significantly more aggregates as previously reported [5], [11], [29]. LRRK2-IN-1 treatment resulted in the relocalization of the wild-type, G2019S and R1441C to more fibrillar-like structures similar to what was reported previously [14], [16]. Interestingly, the localization of kinase dead D1994A was not affected by LRRK2-IN-1 (Figure 1A). Similar localization results were observed for HEK293T, SH-SY5Y cells and primary human astrocytes and for treatment with other LRRK2 inhibitors such as H-1152 and sunitinib (data not shown). To further confirm the expression of full length LRRK2, Western blot analysis was performed using anti-LRRK2 pSer935-specific or anti-pan LRRK2 antibody. Shown in Figure 1B, LRRK2 wild-type, G2019S and to a much lesser extent R1441C showed constitutive phosphorylation at Ser935, which can be diminished by the treatment of LRRK2-IN-1. Minimal phosphorylation of Ser935 was observed for D1994A. We evaluated the phosphorylation of LRRK2 expressed in U-2 OS via BacMam at Ser910, Ser935, Ser955 and Ser973 and found that indeed LRRK2 was modified at these sites and that they were responsive to LRRK2-IN-1 treatment as reported previously in other systems [22] (Figure S1). These results demonstrate that LRRK2-GFP can be efficiently expressed via BacMam gene delivery system and the expressed LRRK2-GFP displayed similar cytoplasmic localization and Ser935 phosphorylation pattern to those reported with N-terminal GFP-LRRK2.

### Development of a TR-FRET Cellular Assay for LRRK2 Ser935 Phosphorylation

**Figure 2:**
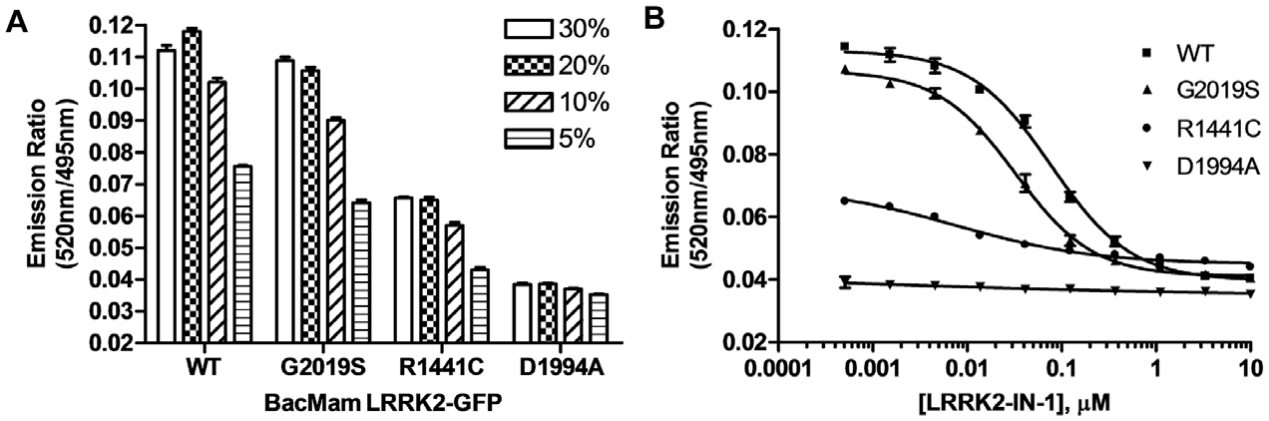
TR-FRET detection of Ser935 phosphorylation of LRRK2-GFP in U-2 OS cells. (A) U-2 OS cells were transduced with indicated amounts of BacMam LRRK2-GFP wild-type, G2019S, R1441C or D1994A. Cells were plated onto a 384-well assay plate and lysed in the presence of Tb-labeled anti-pSer935 antibody. TR-FRET was measured and the emission ratios of 520 nm/490 nm are plotted against the amount of BacMam used for transduction. (B) Cells transduced with 20% BacMam LRRK2-GFP were plated onto a 384-well assay plate and treated with indicated amounts of LRRK2-IN-1 for 90 min. Cells were lysed and analyzed as in (A). Emission ratios of 520 nm/495 nm are plotted against the amount of LRRK2-IN-1. All data points represent the average value (±SD) of 6 replicates.

**Figure 3:**
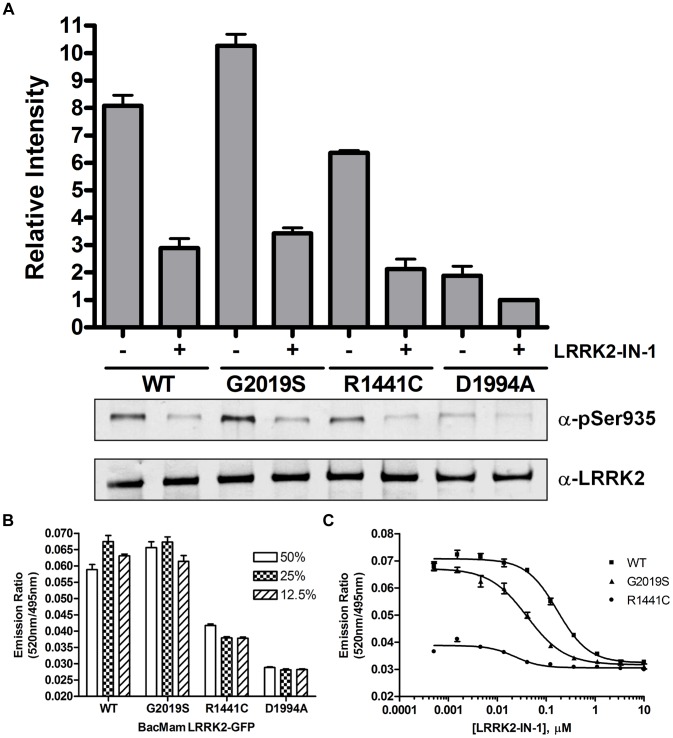
TR-FRET detection of Ser935 phosphorylation of LRRK2-GFP in SH-SY5Y cells. (A) SH-SY5Y cells were transduced with 25% BacMam LRRK2-GFP wild-type, G2019S, R1441C or D1994A. Cells were treated with DMSO only or LRRK2-IN-1 (3 µM) for 90 min. Western blot analysis with anti-pSer935 antibody or anti-LRRK2 antibody was performed. The quantifications are from 3 independent experiments. (B) SH-SY5Y cells were transduced with indicated amounts of BacMam LRRK2-GFP wild-type, G2019S, R1441C and D1994A. Cells were plated onto a 384-well assay plate, lysed and analyzed as described in Figure 2 legend. Emission ratios of 520 nm/495 nm are plotted against the amount of BacMam used for transduction. (C) Cells transduced with 25% BacMam LRRK2-GFP were plated onto a 384-well assay plate and treated with indicated amounts of LRRK2-IN-1 for 90 min. Cells were lysed and analyzed as in (B). Emission ratios of 520 nm/495 nm are plotted against the amount of LRRK2-IN-1. All data points represent the average value (±SD) of 6 replicates.

**Figure 4:**
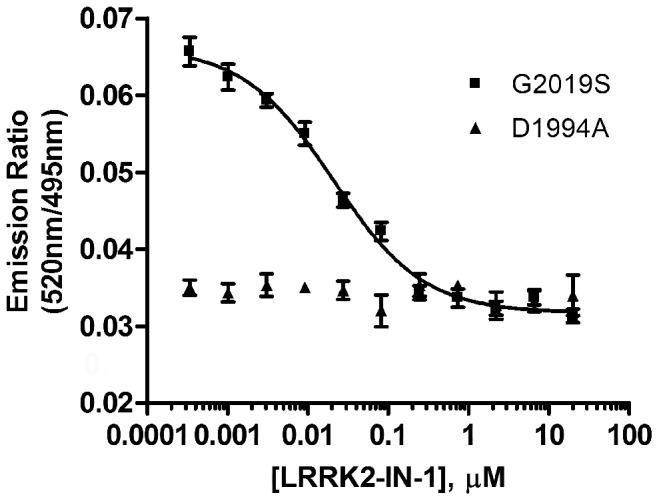
TR-FRET detection of Ser935 phosphorylation of LRRK2-GFP in human neural stem cells. Human neural stem cells were transduced with 10% BacMam LRRK2-GFP G2019S or D1994A. Cells were plated onto a 384-well assay plate and treated with indicated amounts of LRRK2-IN-1 for 90 min. Cells were lysed and analyzed as in Figure 2. Emission ratios of 520 nm/495 nm are plotted against the concentration of LRRK2-IN-1. All data points represent the average value (±SD) of 6 replicates.

The second step for developing a LanthaScreen® TR-FRET cellular assay is to generate terbium labeled modification-specific antibodies. Here, the same antibody that was used in the Western blot analysis for Ser935 phosphorylation (Figure 1B) was labeled with terbium (Tb) as described in **Materials and Methods**. U-2 OS cells were transduced with the indicated concentrations of BacMam LRRK2-GFP wild-type and mutants and plated in a 384-well assay plate. Cells were lysed directly in the assay plate with lysis buffer supplemented with Tb-labeled anti-LRRK2 pSer935 antibody. The presence of phosphorylated Ser935 allows the binding of the antibody to the expressed LRRK2-GFP bringing Tb in close proximity to GFP enabling a FRET to occur from Tb to GFP. The level of phosphorylation at Ser935 can then be quantitatively represented by the TR-FRET signal (the emission ratio of the acceptor GFP to the donor Tb). Shown in Figure 2A, LRRK2-GFP wild-type and G2019S displayed a dose dependent increase of TR-FRET signal with the maximum signal reached at 20% virus used for transduction. R1441C showed significantly lower TR-FRET signal even at 30% virus and D1994A did not display any dose-dependent TR-FRET signal. At 20% concentration, the raw TR-FRET signal generated by wild-type or G2019S is about 2.5 to 3 fold higher than that of D1994A. These results are consistent with the Western blot results in Figure 1B showing differences in the level of Ser935 phosphorylation of LRRK2 wild-type and mutants. To determine whether the TR-FRET assay can quantitatively measure the reported inhibition of Ser935 phosphorylation by LRRK2 kinase inhibitor, U-2 OS cells transduced with 20% of BacMam LRRK2-GFP were incubated with various doses of LRRK2-IN-1 prior to the detection of Ser935 phosphorylation via TR-FRET. Again, D1994A did not display a significant TR-FRET signal whereas R1441C displayed only a low signal. However, wild-type and G2019S transduction resulted in 2.5 fold higher TR-FRET signal which can be inhibited by LRRK2-IN-1 in a dose-dependent manner with IC_50_ values of 0.08 µM and 0.03 µM, respectively (Figure 2B), which is consistent with the IC_50_ values estimated from Western blot results using HEK293 cells stably expressing N-terminal GFP tagged LRRK2 [14]. The successful detection of LRRK2 Ser935 phosphorylation via TR-FRET in U-2 OS cells prompted us to test the feasibility of using difficult-to-transduce, but more relevant cell backgrounds such as neuroblastoma line SH-SY5Y and human neural stem cells (NSCs). After optimizing the transduction protocol, both SH-SY5Y and NSCs were transduced with high efficiency allowing for high level expression of LRRK2-GFP (GFP imaging data not shown). The expression and Ser935 phosphorylation levels of wild-type and mutants in SH-SY5Y cells were confirmed by Western blot analysis (Figure 3A). Consistent with U-2 OS results, the level of Ser935 phosphorylation of LRRK2 wild-type, G2019S and R1441C can be significantly reduced by LRRK2-IN-1 treatment and D1994A displayed little Ser935 phosphorylation. Using the same TR-FRET detection reagents and method as those used for U-2 OS cells, LRRK2 Ser935 phosphorylation of wild-type and G2019S, and to a much lesser extent R1441C were detected via TR-FRET (Figure 3B). In this case, 25% BacMam LRRK2-GFP wild-type and G2019S generated a greater than 2-fold higher TR-FRET signal than D1994A, which displayed background emission ratios similar to those from untransduced cells (data not shown). R1441C generated a TR-FRET signal higher than D1994A, but significantly lower than wild-type and G2019S. Again the TR-FRET signals generated from cells transduced with wild-type and G2019S were inhibited by LRRK2-IN-1 in a dose dependent manner with an IC_50_ values of 0.17 and 0.04 µM, respectively (Figure 3C), which is consistent with the U-2 OS data. LRRK2-IN-1 dose-dependent inhibition of G2019S Ser935 phosphorylation is also observed and measured via TR-FRET using human neural stem cells derived from the NIH approved H9 human embryonic stem cells (Figure 4). The IC_50_ value of LRRK2-IN-1 was calculated to be 0.03 µM, similar to the value obtained in SH-SY5Y cells. D1994A again showed only background emission ratios which was not affected by LRRK2-IN-1 treatment.

### Known Inhibitor Profiling

To further validate the TR-FRET cellular assay, LRRK2-IN-1 and seven other small molecule inhibitors previously reported to have inhibitory activities of LRRK2 kinase were profiled in three different cell backgrounds, U-2 OS, SH-SY5Y and HEK293T transduced with BacMam LRRK2-GFP wild-type or G2019S. The IC_50_ values of these inhibitors are summarized in Table 1. With the exception of H-89 and GW5074, all other 4 known compounds reduced the level of Ser935 phosphorylation of both wild type and G2019S in a dose-dependent manner. Consistent with previous reports using biochemical assays and Western blot analysis, a 1.5- to 3- fold greater sensitivity of G2019S to these inhibitors than wild-type is also detected here by the TR-FRET cellular assay (Table 1). JAK3 Inhibitor VI was shown previously in biochemical TR-FRET binding and activity assays to bind and inhibit LRRK2 wild-type and G2019S with IC_50_ values ranging from 22 nM to 40 nM [23], [30]. Here for the first time, the cellular activity of this compound is reported. It displayed similar IC_50_ values as sunitinib in all three cell backgrounds. ROCK inhibitor H-1152 is less potent than sunitinib, consistent with previous Western blot data [16]. TAE684 inhibited Ser935 phosphorylation of both wild-type and G2019S in all three cell backgrounds with the same potency as LRRK2-IN-1, consistent with previously reported cellular activity [15]. Indirubin-3′-monoxime and GW5074 were reported to inhibit LRRK2 autophosphorylation as well as myelin-basic protein (MBP) phosphorylation by LRRK2 [4]. Here, indirubin-3′-monoxime demonstrated partial inhibitory activity against LRRK2 G2019S Ser935 phosphorylation with micro-molar concentrations (Table 1). Interestingly, GW5074 did not show any activity against LRRK2 wild-type in all three cell backgrounds, but displayed partial inhibitory activity on LRRK2 G2019S in U-2 OS and HEK293T, but not SH-SY5Y cells. H-89 is an inhibitor for protein kinase A which was reported to phosphorylate LRRK2 Ser935 [20], In contrast to the previous *in vitro* report, no effect of H-89 on the phosphorylation of Ser935 was detected in the TR-FRET cellular assay, neither did forskolin (data not shown), a known PKA activator which increases cAMP levels.

**Table 1:** IC_50_ values (µM) of known compounds determined in the TR-FRET cell-based assay for Ser935 phosphorylation of LRRK2.

	U-2 OS		SH-SY5Y		HEK293T	
Compound	WT	G2019S	WT	G2019S	WT	G2019S
LRRK2-IN-I	0.09+/−0.05	0.05+/−0.02	0.20+/−0.03	0.06+/−0.03	0.12+/−0.04	0.06+/−0.03
TAE684	0.09+/−0.04	0.07+/−0.01	0.06+/−0.02	0.03+/−0.01	0.12+/−0.04	0.10+/−0.03
JAK3 Inh. VI	0.57+/−0.19	0.17+/−0.04	2.77+/−0.76	0.68+/−0.07	0.81+/−0.31	0.26+/−0.05
Indirubin-3-monoxime	3.78+/−0.10*	1.93+/−0.09*	>10	6.15+/−2.75*	3.68+/−2.03*	2.78+/−1.10*
Sunitinib	0.29+/−0.07	0.16+/−0.01	0.62+/−0.36	0.32+/−0.07	0.45+/−0.14	0.32+/−0.16
GW5074	>20	0.47+/−0.14*	>20	>20	>20	0.28+/−0.12*
H-1152	3.28+/−0.69	0.89+/−0.05	1.66+/−0.04	0.77+/−0.20	2.94+/−0.98	1.38+/−0.58
H-89	>20	>20	>20	>20	nd	nd

IC_50_ (µM) +/− SD; *Partial inhibition; nd: not tested.

### Tocris Library Screen and Confirmation of Hits

**Figure 5:**
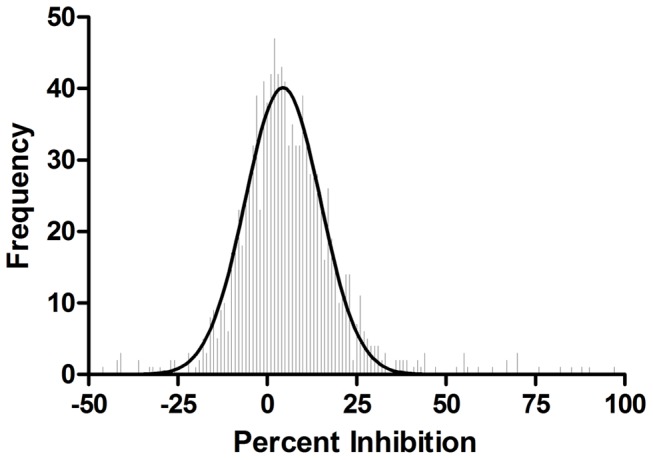
Tocriscreen™ mini library screening results. Average percent inhibition of library compounds was analyzed to produce a frequency distribution with binning equal to one percent increments. The frequency distribution was plotted and a gaussian curve fit to the distribution (curve statistics: mean 4.38% standard, deviation 10.53% and R2 value of 0.962).

To test the performance of this assay in a high-throughput screen, Tocriscreen™ Mini library of 1120 biologically active compounds which target ion channels, G-protein-coupled receptors (GPCRs), nuclear receptors and kinases were screened in the TR-FRET Ser935 phosphorylation cellular assay using BacMam LRRK2-GFP G2019S transduced SH-SY5Y cells. Each compound was tested twice at a single concentration of 20 µM in 0.1% DMSO using duplicate 384-well assay plates, to reproduce actual conditions that may be used in a larger high-throughput screen. LRRK2-IN-1 was included as a positive control on each plate and used at 10 µM. Percent inhibition of each compound is calculated and plotted in a histogram shown in Figure 5. Both sets of plates showed similar results, with an average Z′-factor of 0.75 and an average assay window of 2.1. These data demonstrate the robustness of this assay and its suitability for use in screening applications.

Out of the 1120 compounds, 16 compounds reduced the level of Ser935 phosphorylation by greater than 50%. Among the 16 compounds, SP600125, indirubin-3′-monoxime and ROCK inhibitor Y-27632 with previously reported LRRK2 inhibitory activity showed 88%, 67% and 63% inhibition, respectively in this screen (Table 2 first column). An interesting observation is that 5 out of the 16 compounds (Table 2, Bay 11-7085, Bay 11-7821, IKK16, Ro106-9920 and TPCA-1) were IKK (or NFκB pathway) inhibitors and 5 compounds (Table 2, NSC-95397, PD407824, NSC-663284, aminopurvalanol A and SB 218078) were cell-cycle pathway kinases such as CHK1 or CDC25 phosphatase inhibitors. Besides kinase inhibitors, GPCR ligands such as opioid-receptor like-1 (OPRL-1) antagonist JTC801 (90% inhibition), dopamine receptor D1 antagonist BNTX maleate (70% inhibition), adenosine A2 receptor agonist CV1808 (55% inhibition, data not shown) and dopamine D2-like receptor ligand 3′-fluorobenzylspiperone maleate (53% inhibition, data not shown) also showed up as hits in this assay. These 16 compounds were followed-up in dose-response experiments (Figure S2). Both maximum percent inhibition and IC_50_ values for each compound in the follow-up study are summarized in columns 4 and 5 of Table 2. The maximum percent inhibition in the follow-up study matches well to that of the initial screen. Seven compounds SP600125, IKK16, NSC-95397, GW441756, PD407824, aminopurvalanol A and TPCA-1 displayed low µM IC_50_ values. Several compounds such as JTC-801 and the two NSC compounds displayed steep dose-response curves (Figure S2). To examine the cytotoxic effects of these compounds, SH-SY5Y cells expressing LRRK2-GFP G2019S were treated with these compounds under the same conditions used in the TR-FRET dose-response follow-up study and cell viability was monitored using a resazurin-based cell viability assay. Most compounds tested show little effect on cell viability (Table 2, column 6). Three compounds (Bay 11-7821, NSC-95397 and PD407824) significantly lowered the cell viability at much higher IC_50_ values than those for the TR-FRET assay. Two compounds (JTC-801 and NSC-663284) were toxic at lower concentrations than those needed to in the TR-FRET assay. The two NSC compounds also have strong red color which could potentially affect the TR-FRET readout causing steep dose-response curves.

**Table 2:** Top 16 hits of the Tocriscreen™ Mini library screen.

%Inhibition	Compound	Known Activity	Cellular TR-FRET Assay		Cytotoxicity Assay	Biochemical LRRK2 assay
Initial screen			Max % Inhibition	IC_50_ (µM) +/−SD	IC_50_ (µM)	IC_50_ (µM) +/− SD
97.5	Bay 11-7085	Irreversible inhibitor of TNFa-induced IkB phosphorylation	100	5.3+/−2.8	>20	>100
90.1	JTC 801	ORL1 antagonist	84	9.6+/−6.3	10	>100
88.3	SP 600125	JNK and other kinase inhibitor	103	1.0+/−0.4	>20	0.22+/−0.16
85.5	Bay 11-7821	Irreversible inhibitor of TNFa-induced IkB phosphorylation	94	4.0+/−2.1	20	>100
81.7	IKK 16	Inhibitor of IKK	82	1.3+/−0.8	>20	0.05+/−0.03
76.1	NSC 95397	Selective Cdc25 dual specificity phosphatase inhibitor	80	1.3+/−0.9	7.8	15+/−5.5
69.8	Ro 106-9920	Inhibitor of NFkB activation	76	7.6+/−0.01	20	>100
69.7	GW 441756	TrkA inhibitor	72	2.2+/−0.3	>20	0.32+/−0.18
69.7	BNTX maleate	Standard d1 selective antagonist	88	4.0+/−1.4	>20	>100
67.3	PD 407824	Inhibitor of Chk1 and Wee1	65	0.5+/−0.04	15	0.32+/−0.32
67.3	Y-27632	p160ROCK inhibitor	69	10+/−4.7	>20	0.57+/−0.27
63.5	Indirubin-3-oxime	GSK-3b inhibitor. Also inhibits other protein kinases	64	4.4+/−0.5	>20	>10
59.1	NSC 663284	Cdc25 phosphatase inhibitor	45	5.8+/−1.2	2	0.48+/−0.32
56.2	Aminopurvalanol A	Cyclin-dependent kinase inhibitor	50	1.4+/−2.2	>20	ND
55.3	SB 218078	Inhibitor of checkpoint kinase 1 (Chk1)	54	9.5+/−0.4	>20	ND
54.8	TPCA-1	Inhibitor of IKK-2	50	0.1+/−0.03	>20	ND
100	LRRK2 IN-1	Positive Control Compound	100	0.07+/−0.02	>20	0.01+/−0.001

The IC_50_ values are the averaged numbers from three independent experiments for both cellular and biochemical TR-FRET assays and one representative data of two independent experiments for the cytotoxicity assay.

From these initial screens, we evaluated 13 of these compounds further by determining the *in vitro* activity against LRRK2 in order to make correlations between *cellular* and *in vitro* activities. We observed the previously unreported inhibitors of LRRK2, GW441756, PD407824, IKK16, exhibited *in vitro* IC_50_s of 320 nM, 320 nM and 50 nM, respectively (Table 2, column 7). LRRK2 was inhibited by SP600125 and Y-27632 as was observed previously [4], [5]. SP600125, IKK16, GW441756, Y-27632 and NSC-663284 exhibited sub-micromolar IC_50_ concentrations for LRRK2 *in vitro,* which was lower than what observed for cellular inhibition of Ser935 phosphorylation. PD407824 displayed similar IC_50_ values in the *in vitro* and cellular assays, whereas NSC95397 inhibited *in vitro* kinase activity at higher concentrations than those for cellular Ser935 phosphorylation. Compounds BNTX, JTC801, Bay 11-7085, Bay 11-7821, Ro106-9920 and indirubin-3′-oxime did not repress LRRK2 activity *in vitro*.

**Figure 6:**
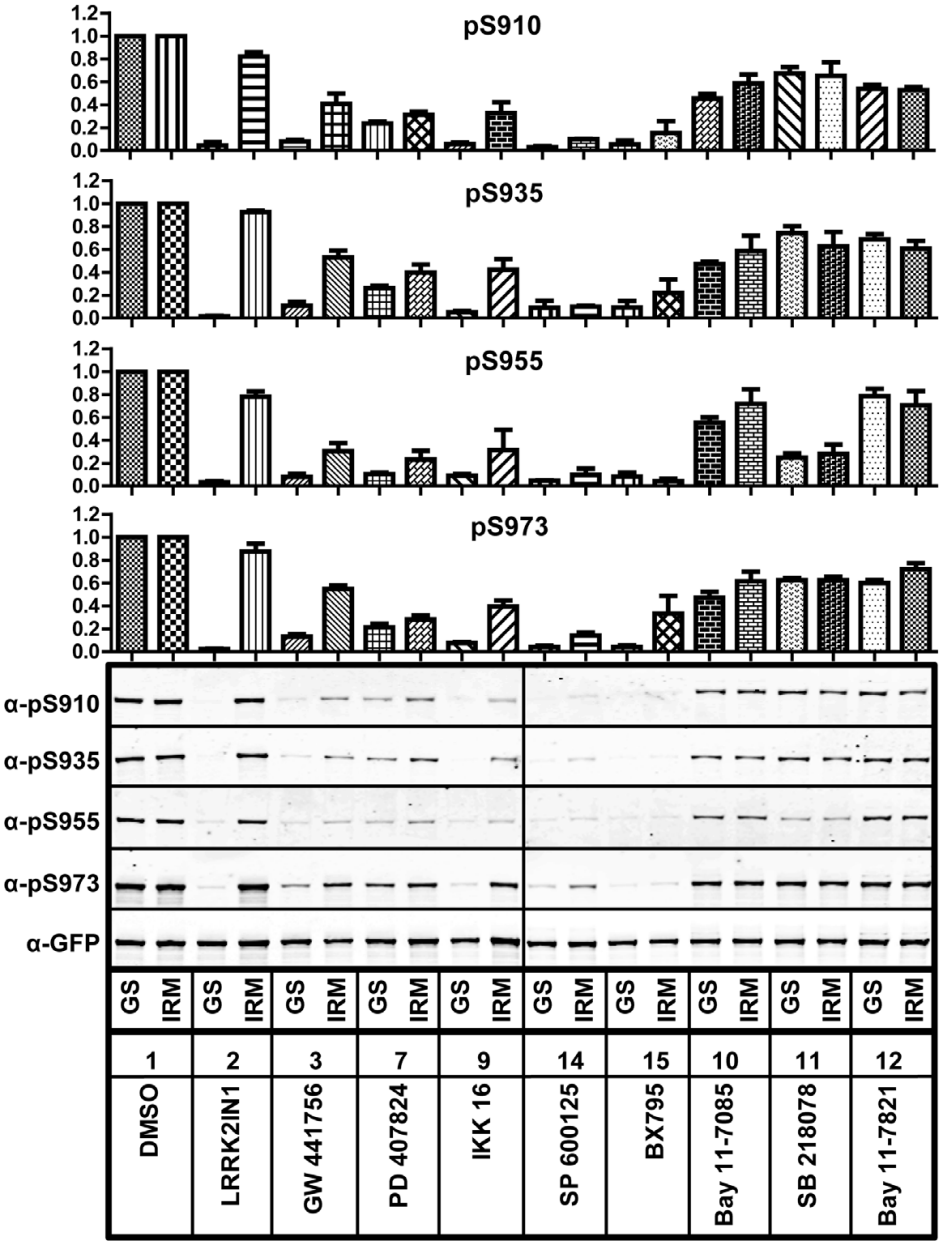
The effects of hit compounds on LRRK2 phosphorylation at serines 910, 935, 955 and 973. Flp-In T-REx™ HEK293 GFP-LRRK2 G2019S cells (GS) or A2016T/G2019S (IRM) were induced and treated with indicated compounds at 20 µM. After cell lysis, GFP-LRRK2 was immunoprecipitated and the phosphorylation of LRRK2 was analyzed by immunoblotting. The quantifications are from 3 independent experiments.

To further confirm the TR-FRET cellular assay results, we also evaluated the effects of these compounds on the cellular phosphorylation of Ser910, Ser935, Ser955 and Ser973 by immunoprecipitation (IP) followed by Western analysis. Here, commonly used HEK293 cells inducibly expressing N-terminal GFP tagged LRRK2 G2019S [5], [14], [16], [21], [22] were used instead of the C-terminal GFP LRRK2 used in the TR-FRET cellular assay. As shown in Figure 6, besides LRRK2-IN-1, several compounds identified as “hits” such as GW441756, PD407824, IKK16 and SP600125 inhibited phosphorylation of Ser910, Ser935, Ser955 and Ser973. These data correlate well with *in vitro* biochemical and cellular TR-FRET data indicating that these may well be direct LRRK2 inhibitors. BNTX maleate, NSC-95397, JTC801 (data not shown), SB218078, BAY11-7805 and BAY11-7821 moderately reduced phosphorylation of all four residues. The observation that NFκB pathway inhibitors modulated LRRK2 kinase activity correlated with unpublished data indicating IKKepsilon and TBK1 phosphorylate LRRK2 *in vitro* (S. Riddle and RJ. Nichols). We therefore employed the kinase inhibitor BX-795, which was first identified as a PDK1 inhibitor, but was subsequently shown to also potently inhibit IKKepsilon and TBK1 [31]. Treatment of cells with this compound revealed that it too inhibited LRRK2 cellular phosphorylation.

We further analyzed the compounds that were found to directly inhibit LRRK2 assays, using the T-REx cellular system expressing an inhibitor desensitizing mutant LRRK2 A2016T/G2019S [5]. This substitution typically increases the IC_50_ value for inhibitors directly acting on LRRK2. We observed that the LRRK2 A2016T/G2019S mutant rescued phosphorylation in the presence of LRRK2-IN-1 treatment as well as with GW44766, IKK16 and BX-795 even at the high concentrations utilized in these assays, revealing that the later three compounds do directly inhibit LRRK2 in cells (Figure 6, IRM). The effect of BAY11-7805, BAY11-7821 and SB218078 did not appear to be changed by the presence of the desensitizing mutation, suggesting that these compounds are not direct LRRK2 inhibitors. Taken together, these Western data indicate the results with cells expressing N-terminally tagged GFP-LRRK2 G2019S correlate well with *in vitro* kinase inhibitory data and confirm the data of the TR-FRET cellular assay using C-terminal GFP tagged LRRK2.

## Discussion

We have developed a high-throughput and homogenous cellular TR-FRET immunoassay for the measurement of LRRK2 phosphorylation. Since acute inhibition of LRRK2 kinase activity can reduce the level of Ser935 phosphorylation, this assay can be applied to high-throughput cell-based screens for LRRK2 kinase inhibitors. Screening of a small molecule inhibitor library with this assay indeed revealed several inhibitors with previously unknown LRRK2 activity as well as provided leads to cellular pathways that could involve LRRK2.

This high-throughput assay utilizes cells expressing full-length human LRRK2 with a C-terminal GFP tag (LRRK2-GFP). We provide multiple lines of evidence suggesting that LRRK2-GFP functions and behaves similarly to the previously reported N-terminally tagged LRRK2 (GFP-LRRK2) stably expressed in HEK293 cells. First, wild-type and G2019S LRRK2-GFP displayed a diffuse cytoplasmic localization [14], [21], [28] and upon LRRK2-IN-1 treatment, a portion of LRRK2 relocalized to more aggregate and fibrillar-like structures similar to was observed previously, where H-1152 treatment of cells induced cytoplasmic accumulation of LRRK2 that appeared to colocolize with microtubules [16]. The nature of these accumulations has yet to be thoroughly investigated but has been widely observed. BacMam mediated expression of LRRK2 R1441C reproduced previous observations that many Roc and COR domain pathogenic mutants induce cytoplasmic accumulations of LRRK2 [21], [29]. Interestingly, acute inhibition of R1441C mutant also induced a redistribution of LRRK2 to more filamentous aggregates similar to what was observed for the Ser910Ala/Ser935Ala mutant [22].

Second, LRRK2-GFP wild-type, G2019S, R1441C and D1994A showed similar Ser935 phosphorylation pattern determined by Western blot and the TR-FRET assay as reported for GFP-LRRK2 [14]. Third, known LRRK2 inhibitors such as LRRK2-IN-1, TAE684, sunitinib and H-1152 inhibited the phosphorylation of Ser935 on LRRK2-GFP wild-type and G2019S to a similar degree and with similar rank order potency as previously reported for GFP-LRRK2. Lastly, other phosphorylation sites such as Ser910, Ser955 and Ser973 reported for N-GFP-tagged LRRK2 can also be phosphorylated on the LRRK2-GFP and inhibited by LRRK2-IN-I (Figure S1).

One of the common methods for detecting LRRK2 phosphorylation is immunoblotting, typically after immunoprecipation. The complexities of this methodology make it highly impractical for the processing and analysis of large sample numbers typically associated with screening experiments. The TR-FRET cellular assay reported here is in a fully homogenous format without washing, lysate transfer, or separation procedures. Cells transduced with BacMam LRRK2-GFP can be plated onto a 384-well assay plate, treated as desired and then incubated with 6 X lysis buffer containing Tb-labeled detection antibody added directly to the wells. To further simplify the procedure, we demonstrated that cells can be transduced, cryopreserved, later thawed and used directly to run the assay without the need for culturing (Figure S3). This indicates that one can perform a large batch transduction and cryopreserve the cells for screening applications at a later time, which will reduce the time, labor and day-to-day variables.

We profiled 1120 compounds of the Tocriscreen™ Mini library to gain insight into the biology of Ser935 phosphorylation and demonstrate the utility of the TR-FRET cellular assay in screening. Using a maximal inhibition threshold of 50%, we observed the previously identified inhibitors of LRRK2 SP600125, indirubin-3′-monoxime and ROCK inhibitor Y-27632 induced dephosphorylation of Ser935, which increased confidence in our assays. In addition to compounds with known LRRK2 inhibitory activity, we identified several novel LRRK2 kinase inhibitors from this screen. GW441756 is a known TrkA inhibitor [32] and has some degree of structure similarity to GW5074. Here we show GW441756 can inhibit the phosphorylation of LRRK2 on Ser935 in cells by both TR-FRET assays and IP-Western. We further confirmed that GW441756 is a LRRK2 kinase inhibitor in the TR-FRET LRRK2 *in vitro* kinase assay with an IC_50_ of 320 nM. Interestingly, we did not observe *in vivo* dephosphorylation of Ser935 with the PKC inhibitors GF109203X and Ro31-8220, which were present in the Tocriscreen library and identified previously to inhibit LRRK2 autophosphorylation *in vitro* [4].

Interestingly, we observed that 5 out of the top 16 compounds Bay 11-7085, Bay 11-7821, IKK16, Ro106-9920 and TPCA-1 intersect with the NFκB pathway. TPCA-1 and IKK16 are IKK inhibitors, however LRRK2 is inhibited by IKK16 at an *in vitro* IC_50_ 20 times lower than the cellular IC_50_, indicating that the cellular effect may be due to LRRK2 inhibition. Ro106-9920 inhibits IκB degradation and LPS induced cytokine (TNF-alpha, IL1-beta and IL-6) production [33] and suppressed LRRK2 phosphorylation on Ser935 by 77% in SH-SY5Y cells and approximately 50% in HEK293 cells. Bay 11-7085 and Bay 11-7821 block NFκB activation by inhibiting the IκB complex. In 293 cells, the inhibitors Bay 11-7085 and Bay 11-7821 consistently induced dephosphorylation of both LRRK2 G2019S and G2019S/A2016T to 50% of untreated G2019S indicating an independence of direct action on LRRK2. These data indicate that IKKβ is a potential *in vivo* kinase regulating LRRK2 phosphorylation. The inhibitor BX-795, which inhibits PDK1 as well as the TBK1 and IKKε kinases, decreased the phosphorylation of the cellular phosphorylation sites in HEK293 T-REx™ cells expressing GFP-LRRK2 G2019S, and the G2019S mutation showed decreased sensitivity to BX795 indicating it is indeed a direct inhibitor of LRRK2. BX795 treatment cannot then be utilized to rule in or out IKKε or TBK1 in their ability to phosphorylate LRRK2 *in vivo*. Interestingly, inhibition of LRRK2 Ser935 phosphorylation occurs in the absence of receptor stimulation and may represent perturbing a basal status of modification. Together, these data indicate that the pathways that induce NFκB activation play a role in LRRK2 phosphorylation, but this regulation is likely complex.

Inhibitors of the Cdc25 phosphatase NSC-95397 and NSC-663284 or kinase inhibitors aminopurvalanol A (CDK), SB 218078 and PD407824 (CHK1) can block cell cycle progression [34], [35], [36] and were found to also repress LRRK2 Ser935 phosphorylation. PD407824 inhibited LRRK2 *in vitro* and Ser935 phosphorylation in cells indicating that indeed this is potentially another direct LRRK2 kinase inhibitor. Since the LRRK2 inhibitor resistant mutant did not show decreased sensitivity to this compound, it may well be that CHK1 could act on the cellular phosphorylation sites in a LRRK2 kinase activity independent manner. In the future, it would be interesting to investigate LRRK2 activity and phosphorylation during different stages of the cell cycle, especially since there are multiple lines of evidence linking LRRK2 to cancer [37], [38].

We also observed compounds other than kinase inhibitors that affect LRRK2 phosphorylation, but do not directly target LRRK2 enzyme itself, namely GPCR ligands such as opioid-receptor like-1 (OPRL-1) antagonist JTC801, the dopamine receptor D1 antagonist BNTX maleate, dopamine D2-like receptor ligand 3′-Fluorobenzylspiperone maleate, and an agonist of the adenosine A2 receptor CV1808. Though results are complicated for some of these compounds due to their cytotoxic effects, these compounds could indicate potential signaling pathways that also alter LRRK2 function via effects on LRRK2 phosphorylation. These represent avenues for further research.

In summary, we have developed a robust high-throughput detection system for evaluating the phosphorylation of LRRK2 at Ser935 that correlates well with alternate systems. A small library was used to evaluate this screening system and hits from that screen were validated in established systems showing the reliability of the methodology. By exploiting the biology of Ser935 phosphorylation, we gained novel insights into potential pathways that intersect with LRRK2, as well as identified other possible direct LRRK2 inhibitors.

## Materials and Methods

ReagentsAll cell culture media and supplements, BacMam LRRK2-GFP reagents, PrestoBlue™ cell viability assay reagent, Western blotting reagents and human neural stem cells were obtained from Life Technologies (Carlsbad, CA). All other cell lines were purchased from ATCC (Manassas, VA). The cell lines were maintained under conditions recommended by the manufacturer. Anti-LRRK2 antibody used for Western blotting was purchased from Cell Signaling Technologies (Danvers, MA). Protease and phosphatase inhibitor cocktails and H-89 were purchased from Sigma (St. Louis, MO). LRRK2-IN-1 was provided by the Michael J. Fox Foundation for Parkinson’s Research. Sunitinib was from LC Laboratories (Woburn, MA), JAK3 Inhibitor VI and Indirubin-3′-monoxime were from EMD Biosciences (San Diego, CA). H-1152, GW5074 and the Tocriscreen™ Mini library and the follow-up compounds were from Tocris (Ellisville, MO). Rabbit polyclonal anti-phospho Ser910, Ser935, Ser955 and Ser973 antibodies were produced by Yenzym Antibodies (South San Francisco, CA) as described in [22]. The anti-LRRK2 pSer935 antibody was labeled with amine-reactive terbium (Tb)-chelate (Life Technologies) according to manufacturer’s protocol. Un-reacted Tb-chelate was removed from the solution via dialysis in HBS (pH7.5). The antibody concentration and degree of labeling was determined by absorbance measurements.

### Cell Culture and BacMam Transduction

U-2 OS cells are maintained in growth medium (McCoy’s 5A supplemented with 10% dialyzed FBS (dFBS), 10 mM HEPES, 0.1 mM NEAA, 1 mM Sodium Pyruvate, and 100 U/mL Penicillin/100 µg/mL Streptomycin). HEK293T cells are maintained in DMEM with 10% dFBS, 10 mM HEPES, 0.1 mM NEAA and 100 U/mL Penicillin/100 µg/mL Streptomycin. SH-SY5Y cells are maintained in DMEM/F-12 medium supplemented with 10% dFBS and 100 U/mL Penicillin/100 µg/mL Streptomycin. HEK293 T-REx™ GFP-LRRK2 G2019S cells described in [22] were maintained in DMEM as above (except NEAA) and with 10 µg/ml Blasticidin and 50 µg/ml Hygromycin. GIBCO® human neural stem cells (hNSCs) were propagated as an adherent culture in StemPro® NSC SFM according to the manufacturer’s recommended protocol (Life Technologies). For BacMam transduction of U-2 OS and HEK293T, cells were harvested, mixed with various amounts of BacMam LRRK2-GFP reagent (v/v) and plated onto 6-well plates. The following day, cells were harvested, plated onto 384-well assay plates (Corning, MA, #3570, 20 µL/well, about 10,000 cells/well) and incubated overnight. For the transduction of SH-SY5Y cells, various amounts of BacMam LRRK2-GFP reagent (25% or indicated, v/v) are added to cells already grown on a 6-well plate. Cells were assayed in 384-well format (∼20,000 cells/well) 48 hours post-transduction as above. The hNSCs were transduced with either BacMam LRRK2-GFP G2019S or D1994A (10% v/v) overnight and then replated in a 384-well plate in growth medium at a density of ∼3,000 cells/well for assay the following day.

### TR-FRET Cellular Assay

Transduced cells in the 384-well assay plate were incubated with indicated inhibitors (5X, 5 µL/well) for 60 to 90 minutes. During this time, 6 X lysis buffer (Life Technologies) was supplemented with 30 nM Tb-labeled anti-LRRK2 pSer935 antibody and both protease and phosphatase inhibitor cocktails (Sigma). The complete 6 X lysis buffer was added to each well (5 µL/well) and the plates were incubated at room temperature in the dark for 2 hours. The plate was then read on an EnVision® multilabel plate reader (PerkinElmer, Waltham, MA) with excitation at 340 nm and emission 520 nm and 495 nm. Emission ratios of 520 nm/495 nm were calculated and plotted against the concentration of compound. A sigmoidal dose-response equation with varying slope was used to fit the data and generate IC_50_ values. Z′-factor values were calculated as: Z′-factor  = 1 - [(3 × stdev_no virus_ +3 × stdev_maxvirus_)/(avg_maxvirus_ – avg_no-virus_)]. The IC_50_ values reported in Table 1 are the averaged numbers from three independent experiments.

### Tocriscreen™ Mini Library Screen

SH-SY5Y cells were transduced with 25% (v/v) BacMam LRRK2-GFP G2019S and plated (20 µL/well, 20,000 cells/well) onto eight 384-well assay plates as described above. 40 nL of 10 mM Tocris library compounds in DMSO were dispensed into each well of the assay plate by the ECHO system. Equal volumes of DMSO were dispensed into the control wells. Cells were incubated with compounds for 90 to 120 min prior to cell lysis and detection as described above. The percent inhibition is calculated as: [(average emission ratios of DMSO control wells – average emission ratios of compound wells)/(average emission ratios of DMSO control wells – average emission ratios of LRRK2-IN-1 treated wells)] * 100. For the dose-response follow-up studies, fresh compound DMSO stock solutions were prepared from powder. Curve fitting and IC_50_ value calculations were performed using XLFit4 software (IDBS) and a nonlinear regression equation for variable-slope sigmoidal dose response (model number 205). Z′-factor value was calculated as: Z′-factor  = 1 - [(3 × stdev_LRRK2-IN-1_+3 × stdev_DMSO_)/(avg_DMSO_ – avg_LRRK2-IN-1_)]. The IC_50_ values reported in Table 2 are the averaged numbers from three independent experiments for both cellular and biochemical TR-FRET assays and one representative result of two independent experiments for the cytotoxicity assay.

### Fluorescence Microscopy

U-2 OS cells were transduced with BacMam LRRK2-GFP as described above. The cells were then plated on chambered coverslips (LabTek II/Nunc, Rochester, NY) at 50% confluency and incubated for 48 hours. The growth medium was replaced with phenol-red free DMEM® (Life Technologies, Carlsbad, CA) before live imaging. Cells were imaged under the same conditions for each mutant using an inverted microscope (Nikon Eclipse TE2000-S) equipped with a PlanApo 60X objective and a charge-coupled device camera (Diagnostic Instruments SPOT RT 2.3.0) using SPOT Advanced software.

### Immunoprecipitation and Immunoblot Analysis

Immunoblot analysis: BacMam LRRK2-GFP transduced U-2 OS or SH-SY5Y cells (10^7^ cells) were left untreated or treated with 3 µM LRRK2-IN-I for 90 min. For lysate preparation, the cells were washed once with ice-cold PBS and lysed by addition of ice-cold lysis buffer (20 mM Tris-HCl, pH 7.4, 1% NP-40, 5 mM EDTA, 5 mM NaF, 150 mM NaCl, and 1∶100 of protease and phosphatase inhibitor cocktails). The lysate was cleared of debris by centrifugation. Equal amount of total protein was loaded onto each lane for SDS-PAGE (3–8% Tris Acetate). The proteins were transferred onto nitrocellulose membrane using the iBLOT® dry blotting system (Life Technologies) according to manufacturer’s protocol. The membranes were blocked and incubated with a primary antibody against LRRK2 (Cell Signaling Technologies), followed by incubation with alkaline phosphatase-conjugated secondary antibody (WesternBreeze® chromogenic Kit, Life Technologies). The blot was developed by using the chromogenic BCIP/NBT substrate (WesternBreeze® chromogenic Kit, Life Technologies). Immunoprecipitation analysis: HEK293 cells were induced to express GFP-LRRK2 G2019S by inclusion of doxycycline in the culture media at 1 µg/mL. Two days post induction, cells were treated for 90 minutes in the presence of 20 µM of compound or DMSO vehicle control. Cells were rapidly lysed in ice cold lysis buffer 50 mM Tris/HCl, pH 7.5, 1 mM EGTA, 1 mM EDTA, 1% (w/v) 1 mM sodium orthovanadate, 10 mM sodium beta-glycerolphosphate, 50 mM NaF, 5 mM sodium pyrophosphate, 0.27 M sucrose, 1 mM benzamidine and 2 mM phenylmethanesulphonylfluoride (PMSF) and one tablet each complete protease inhibitor and PhosSTOP phosphatase inhibitor (Roche) and was supplemented with 1% (v/v) Triton X-100. GFP-LRRK2 was immunoprecipitated with GFP nanotrap beads (ChromoTek). Immunecomplexes were washed twice with lysis buffer supplemented with 300 mM NaCl and once with Buffer A (50 mM Tris/HCl, pH 7.5, 50 mM NaCl, 0.1 mM EGTA and 0.1% (v/v) 2-mercaptoethanol, and 0.27 M sucrose). Equal amounts of immunoprecipitated LRRK2 were fractionated on 4–12% Novex® NuPAGE® Bis-Tris gels and proteins were transferred to nitrocellulose. Blots were probed with phosphoserine 910, 935, 955 and 973 antibodies as described previously [22].

### *In vitro* Biochemical LRRK2 Activity Assay

The Biochemical LRRK2 TR-FRET activity assay was performed according to the manufacture recommended assay protocol (Life Technologies). Briefly, kinase reactions were performed in 10 µL volumes in black, low-volume 384-well plates (Corning #3676), with 400 nM Fluorescein-ERM (LRRKtide) substrate and 1% DMSO (residual from compound dilutions), in Kinase Buffer S (Life Technologies) supplemented with 2 mM DTT. Reactions performed with ATP equal to K_m,app_ (134 µM) and with 580 ng/mL LRRK2 G2019S (Life Technologies). After incubation at room temperature for 1 hour, the kinase reactions were stopped by addition of 10 µL of 20 mM EDTA and 4 nM Tb-anti-pERM (pLRRKtide) antibody in TR-FRET dilution buffer (Life Technologies). After a 30 minute incubation at room temperature, TR-FRET measurements were obtained on a BMG PHERAstar plate reader using LanthaScreen® filters and settings. A 16 point, 2-fold dilution series of each compound was tested in triplicate at each concentration and assays were also performed in triplicate.

## Supporting Information

Figure S1 **C-GFP tagged LRRK2 phosphorylation at Ser935, Ser955 and Ser973.** U-2 OS cells were transduced with BacMam LRRK2-GFP G2019S and left untreated (−) or treated with LRRK2-IN-1 at 10 µM (+). After cell lysis, the phosphorylation of LRRK2 was analyzed by immunoblotting with indicated antibodies.
(TIF)

Figure S2 **Dose response curves of hit compounds in the TR-FRET cellular assay for LRRK2 Ser935 phosphorylation.** 25% BacMam LRRK2-GFP G2019S transduced SH-SY5Y cells were incubated with indicated concentrations of indicated compounds for 90 min prior to the TR-FRET detection with Tb-anti-LRRK2 pSer935 antibody. The % inhibition is calculated as described in Materials and Methods and plotted against the concentrations of the compounds.
(TIF)

Figure S3 **Dose response curve of LRRK2-IN-1 in the TR-FRET cellular assay using cryopreserved pretransduced SH-SY5Y cells.** SH-SY5Y cells were transduced with 25% BacMam LRRK2-GFP G2019S and then cryopreserved. Cryopreserved cells were thawed, resuspended in assay medium and plated onto 384-well assay plate for 20 hours. Cells were incubated with indicated concentrations of LRRK2-IN-1 for 90 min prior to the TR-FRET detection with Tb-anti-LRRK2 pSer935 antibody. The raw emission ratios are plotted against the concentration of LRRK2. The IC_50_ using cryopreserved cells is about 40 nM, similar to the one generated using freshly transduced cells.
(TIF)

## References

[1] Healy DG, Falchi M, O'Sullivan SS, Bonifati V, Durr A, et al (2008) Phenotype, genotype, and worldwide genetic penetrance of LRRK2-associated Parkinson's disease: a case-control study. Lancet Neurol 7: 583–590. 18539534 10.1016/S1474-4422(08)70117-0 PMC2832754

[2] Cookson MR (2010) The role of leucine-rich repeat kinase 2 (LRRK2) in Parkinson's disease. Nat Rev Neurosci 11: 791–797. 21088684 10.1038/nrn2935 PMC4662256

[3] Covy JP, Giasson BI (2009) Identification of compounds that inhibit the kinase activity of leucine-rich repeat kinase 2. Biochem Biophys Res Commun 378: 473–477. 19027715 10.1016/j.bbrc.2008.11.048 PMC2633649

[4] Lee BD, Shin JH, VanKampen J, Petrucelli L, West AB, et al (2010) Inhibitors of leucine-rich repeat kinase-2 protect against models of Parkinson's disease. Nat Med 16: 998–1000. 20729864 10.1038/nm.2199 PMC2935926

[5] Nichols RJ, Dzamko N, Hutti JE, Cantley LC, Deak M, et al (2009) Substrate specificity and inhibitors of LRRK2, a protein kinase mutated in Parkinson's disease. Biochem J 424: 47–60. 19740074 10.1042/BJ20091035 PMC3759966

[6] Guo L, Gandhi PN, Wang W, Petersen RB, Wilson-Delfosse AL, et al (2007) The Parkinson's disease-associated protein, leucine-rich repeat kinase 2 (LRRK2), is an authentic GTPase that stimulates kinase activity. Exp Cell Res 313: 3658–3670. 17706965 10.1016/j.yexcr.2007.07.007 PMC2083285

[7] Lewis PA, Greggio E, Beilina A, Jain S, Baker A, et al (2007) The R1441C mutation of LRRK2 disrupts GTP hydrolysis. Biochem Biophys Res Commun 357: 668–671. 17442267 10.1016/j.bbrc.2007.04.006 PMC1939973

[8] West AB, Moore DJ, Biskup S, Bugayenko A, Smith WW, et al (2005) Parkinson's disease-associated mutations in leucine-rich repeat kinase 2 augment kinase activity. Proc Natl Acad Sci U S A 102: 16842–16847. 16269541 10.1073/pnas.0507360102 PMC1283829

[9] West AB, Moore DJ, Choi C, Andrabi SA, Li X, et al (2007) Parkinson's disease-associated mutations in LRRK2 link enhanced GTP-binding and kinase activities to neuronal toxicity. Hum Mol Genet 16: 223–232. 17200152 10.1093/hmg/ddl471

[10] Smith WW, Pei Z, Jiang H, Dawson VL, Dawson TM, et al (2006) Kinase activity of mutant LRRK2 mediates neuronal toxicity. Nat Neurosci 9: 1231–1233. 16980962 10.1038/nn1776

[11] Greggio E, Jain S, Kingsbury A, Bandopadhyay R, Lewis P, et al (2006) Kinase activity is required for the toxic effects of mutant LRRK2/dardarin. Neurobiol Dis 23: 329–341. 16750377 10.1016/j.nbd.2006.04.001

[12] Plowey ED, Cherra SJ 3rd, Liu YJ, Chu CT (2008) Role of autophagy in G2019S-LRRK2-associated neurite shortening in differentiated SH-SY5Y cells. J Neurochem 105: 1048–1056. 18182054 10.1111/j.1471-4159.2008.05217.x PMC2361385

[13] Ramsden N, Perrin J, Ren Z, Lee BD, Zinn N, et al (2011) Chemoproteomics-Based Design of Potent LRRK2-Selective Lead Compounds That Attenuate Parkinson's Disease-Related Toxicity in Human Neurons. ACS Chem Biol 6: 1021–1028. 21812418 10.1021/cb2002413 PMC3688284

[14] Deng X, Dzamko N, Prescott A, Davies P, Liu Q, et al (2011) Characterization of a selective inhibitor of the Parkinson's disease kinase LRRK2. Nat Chem Biol 7: 203–205. 21378983 10.1038/nchembio.538 PMC3287420

[15] Zhang J, Deng X, Choi HG, Alessi DR, Gray NS (2012) Characterization of TAE684 as a potent LRRK2 kinase inhibitor. Bioorg Med Chem Lett 22: 1864–1869. 22335897 10.1016/j.bmcl.2012.01.084 PMC3433743

[16] Dzamko N, Deak M, Hentati F, Reith AD, Prescott AR, et al (2010) Inhibition of LRRK2 kinase activity leads to dephosphorylation of Ser(910)/Ser(935), disruption of 14-3-3 binding and altered cytoplasmic localization. Biochem J 430: 405–413. 20659021 10.1042/BJ20100784 PMC3631100

[17] Greggio E, Taymans JM, Zhen EY, Ryder J, Vancraenenbroeck R, et al (2009) The Parkinson's disease kinase LRRK2 autophosphorylates its GTPase domain at multiple sites. Biochem Biophys Res Commun 389: 449–454. 19733152 10.1016/j.bbrc.2009.08.163 PMC2759846

[18] Kamikawaji S, Ito G, Iwatsubo T (2009) Identification of the autophosphorylation sites of LRRK2. Biochemistry 48: 10963–10975. 19824698 10.1021/bi9011379

[19] Webber PJ, Smith AD, Sen S, Renfrow MB, Mobley JA, et al (2011) Autophosphorylation in the leucine-rich repeat kinase 2 (LRRK2) GTPase domain modifies kinase and GTP-binding activities. J Mol Biol 412: 94–110. 21806997 10.1016/j.jmb.2011.07.033 PMC3158845

[20] Li X, Wang QJ, Pan N, Lee S, Zhao Y, et al (2011) Phosphorylation-dependent 14-3-3 binding to LRRK2 is impaired by common mutations of familial Parkinson's disease. PLoS One 6: e17153. 21390248 10.1371/journal.pone.0017153 PMC3046972

[21] Nichols RJ, Dzamko N, Morrice NA, Campbell DG, Deak M, et al (2010) 14-3-3 binding to LRRK2 is disrupted by multiple Parkinson's disease-associated mutations and regulates cytoplasmic localization. Biochem J 430: 393–404. 20642453 10.1042/BJ20100483 PMC2932554

[22] Doggett EA, Zhao J, Mork CN, Hu D, Nichols RJ (2012) Phosphorylation of LRRK2 serines 955 and 973 is disrupted by Parkinson's disease mutations and LRRK2 pharmacological inhibition. J Neurochem 120: 37–45. 22004453 10.1111/j.1471-4159.2011.07537.x

[23] Reichling LJ, Riddle SM (2009) Leucine-rich repeat kinase 2 mutants I2020T and G2019S exhibit altered kinase inhibitor sensitivity. Biochem Biophys Res Commun 384: 255–258. 19397894 10.1016/j.bbrc.2009.04.098

[24] Jaleel M, Nichols RJ, Deak M, Campbell DG, Gillardon F, et al (2007) LRRK2 phosphorylates moesin at threonine-558: characterization of how Parkinson's disease mutants affect kinase activity. Biochem J 405: 307–317. 17447891 10.1042/BJ20070209 PMC1904520

[25] Karaman MW, Herrgard S, Treiber DK, Gallant P, Atteridge CE, et al (2008) A quantitative analysis of kinase inhibitor selectivity. Nat Biotechnol 26: 127–132. 18183025 10.1038/nbt1358

[26] Lebakken CS, Riddle SM, Singh U, Frazee WJ, Eliason HC, et al (2009) Development and applications of a broad-coverage, TR-FRET-based kinase binding assay platform. J Biomol Screen 14: 924–935. 19564447 10.1177/1087057109339207

[27] Robers MB, Horton RA, Bercher MR, Vogel KW, Machleidt T (2008) High-throughput cellular assays for regulated posttranslational modifications. Anal Biochem 372: 189–197. 17961489 10.1016/j.ab.2007.09.012

[28] Alegre-Abarrategui J, Christian H, Lufino MM, Mutihac R, Venda LL, et al (2009) LRRK2 regulates autophagic activity and localizes to specific membrane microdomains in a novel human genomic reporter cellular model. Hum Mol Genet 18: 4022–4034. 19640926 10.1093/hmg/ddp346 PMC2758136

[29] Kett LR, Boassa D, Ho CC, Rideout HJ, Hu J, et al (2012) LRRK2 Parkinson disease mutations enhance its microtubule association. Hum Mol Genet 21: 890–899. 22080837 10.1093/hmg/ddr526 PMC3263991

[30] Anand VS, Reichling LJ, Lipinski K, Stochaj W, Duan W, et al (2008) Investigation of leucine-rich repeat kinase 2 Enzymological properties and novel assays. FEBS J 276: 466–478. 10.1111/j.1742-4658.2008.06789.x 19076219

[31] Bain J, Plater L, Elliott M, Shpiro N, Hastie CJ, et al (2007) The selectivity of protein kinase inhibitors: a further update. Biochem J 408: 297–315. 17850214 10.1042/BJ20070797 PMC2267365

[32] Wood ER, Kuyper L, Petrov KG, Hunter RN 3rd, Harris PA, et al (2004) Discovery and in vitro evaluation of potent TrkA kinase inhibitors: oxindole and aza-oxindoles. Bioorg Med Chem Lett 14: 953–957. 15013000 10.1016/j.bmcl.2003.12.002

[33] Swinney DC, Xu YZ, Scarafia LE, Lee I, Mak AY, et al (2002) A small molecule ubiquitination inhibitor blocks NF-kappa B-dependent cytokine expression in cells and rats. J Biol Chem 277: 23573–23581. 11950839 10.1074/jbc.M200842200

[34] Han Y, Shen H, Carr BI, Wipf P, Lazo JS, et al (2004) NAD(P)H:quinone oxidoreductase-1-dependent and -independent cytotoxicity of potent quinone Cdc25 phosphatase inhibitors. J Pharmacol Exp Ther 309: 64–70. 14718602 10.1124/jpet.103.059477

[35] Leung-Pineda V, Ryan CE, Piwnica-Worms H (2006) Phosphorylation of Chk1 by ATR is antagonized by a Chk1-regulated protein phosphatase 2A circuit. Mol Cell Biol 26: 7529–7538. 17015476 10.1128/MCB.00447-06 PMC1636880

[36] Azorsa DO, Gonzales IM, Basu GD, Choudhary A, Arora S, et al (2009) Synthetic lethal RNAi screening identifies sensitizing targets for gemcitabine therapy in pancreatic cancer. J Transl Med 7: 43. 19519883 10.1186/1479-5876-7-43 PMC2702280

[37] Looyenga BD, Furge KA, Dykema KJ, Koeman J, Swiatek PJ, et al (2011) Chromosomal amplification of leucine-rich repeat kinase-2 (LRRK2) is required for oncogenic MET signaling in papillary renal and thyroid carcinomas. Proc Natl Acad Sci U S A 108: 1439–1444. 21220347 10.1073/pnas.1012500108 PMC3029686

[38] Saunders-Pullman R, Barrett MJ, Stanley KM, Luciano MS, Shanker V, et al (2010) LRRK2 G2019S mutations are associated with an increased cancer risk in Parkinson disease. Mov Disord 25: 2536–2541. 20818610 10.1002/mds.23314 PMC2978749

