# Supplementary figures and images for: Screening for Novel LRRK2 Inhibitors Using a High-Throughput TR-FRET Cellular Assay for LRRK2 Ser935 Phosphorylation
Source: PLoS One. 2012 Aug 28;7(8):e43580. doi: 10.1371/journal.pone.0043580 (PMC3429506; doi:10.1371/journal.pone.0043580)

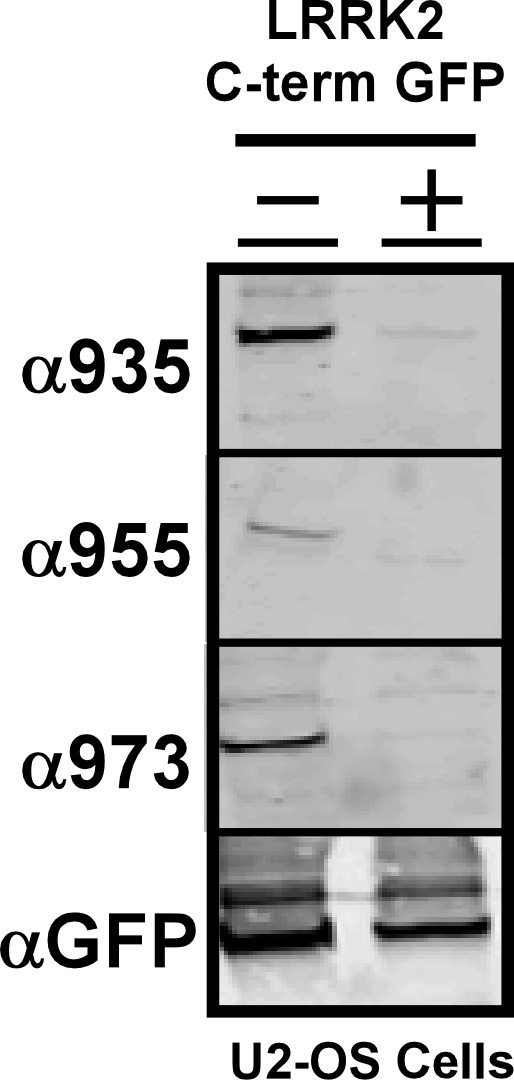

Supplement: Figure S1 — C-GFP tagged LRRK2 phosphorylation at Ser935, Ser955 and Ser973. U-2 OS cells were transduced with BacMam LRRK2-GFP G2019S and left untreated (−) or treated with LRRK2-IN-1 at 10 µM (+). After cell lysis, the phosphorylation of LRRK2 was analyzed by immunoblotting with indicated antibodies. (TIF) [file pone.0043580.s001.tif]

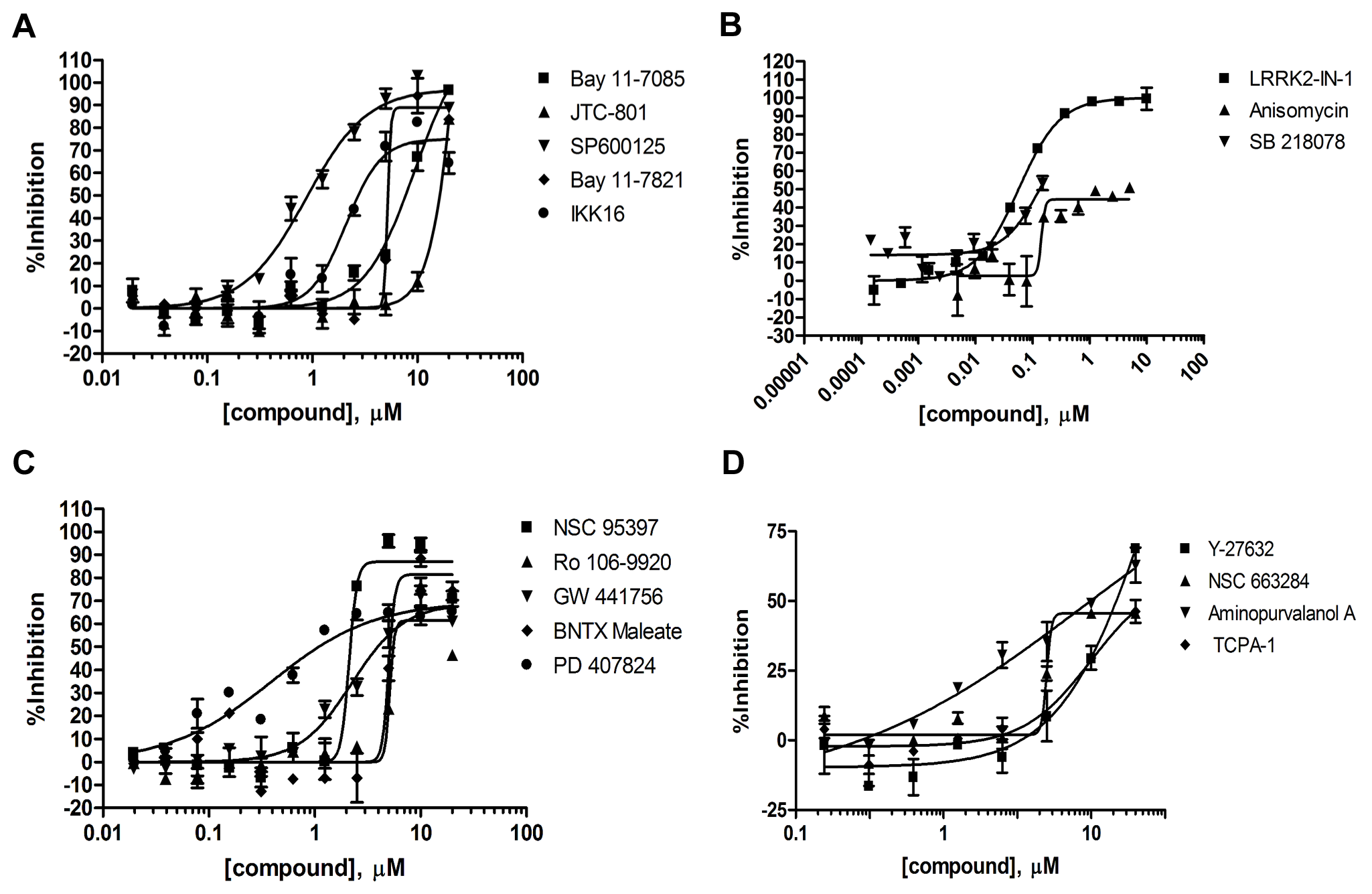

Supplement: Figure S2 — Dose response curves of hit compounds in the TR-FRET cellular assay for LRRK2 Ser935 phosphorylation. 25% BacMam LRRK2-GFP G2019S transduced SH-SY5Y cells were incubated with indicated concentrations of indicated compounds for 90 min prior to the TR-FRET detection with Tb-anti-LRRK2 pSer935 antibody. The % inhibition is calculated as described in Materials and Methods and plotted against the concentrations of the compounds. (TIF) [file pone.0043580.s002.tif]
